# Understanding influencing attributes of COVID-19 vaccine preference and willingness-to-pay among Chinese and American middle-aged and elderly adults: A discrete choice experiment and propensity score matching study

**DOI:** 10.3389/fpubh.2023.1067218

**Published:** 2023-03-16

**Authors:** Xialei Li, Liujun Yang, Guanghua Tian, Bojunhao Feng, Xiaocen Jia, Zonglin He, Taoran Liu, Xianqi Zhao, Mengjie Huang, Wenli Yu, Lian Yu, Wai-Kit Ming

**Affiliations:** ^1^School of Pharmaceutical Sciences, Shandong University, Jinan, China; ^2^School of Public Administration, Renmin University of China, Beijing, China; ^3^Department of Public Health and Health Management, Clinical College of Anhui Medical University, Hefei, China; ^4^Faculty of Medicine, Macau University of Science and Technology, Macau, China; ^5^School of Public Health, Qingdao University, Qingdao, China; ^6^International School, Jinan University, Guangzhou, China; ^7^Department of Public Health and Preventive Medicine, School of Medicine, Jinan University, Guangzhou, China; ^8^School of Public Health, Shandong University, Jinan, China; ^9^School of Liberal Education, Weifang University of Science and Technology, Weifang, China; ^10^School of Public Health, Xi'an Jiaotong University Health Science Center, Xi'an, China

**Keywords:** COVID-19 vaccine, middle-aged and elderly adults, vaccine acceptance, vaccine preference, willingness to pay, public health

## Abstract

**Background and objective:**

COVID-19 has imposed burdens on public health systems globally. Owing to the urgency of vaccination, this study aimed at comparing the differences in preference and willingness to pay of COVID-19 vaccine among Chinese and American middle-aged and elderly adults.

**Methods:**

A cross-sectional survey containing demographic questions, rating their acceptance of COVID-19 vaccination with and without recommendations from friends, family members or employers (the social cues referred to in our study), and a discrete choice experiment understanding COVID-19 vaccine preference and willingness to pay was conducted to collect data. Propensity score matching was utilized to adjust confounding factors of baseline characteristics and the relative importance of respondents' preference for each attribute and its level was estimated using a conditional logit model. Then, willingness to pay was calculated.

**Results:**

In total, 3,494 (2,311 and 1,183 from China and the United States, respectively) completed the questionnaire, among which 3,444 questionnaires were effective. After propensity score matching, 1,604 respondents with 802 from the US and 802 from China were included. Under the influence of the social cues, Chinese respondents' vaccine acceptance decreased from 71.70 to 70.70%, while American respondents' vaccine acceptance increased from 74.69 to 75.81%. The discrete choice experiment showed that American respondents regarded the efficacy of COVID-19 vaccine as the most important attribute, whereas Chinese respondents attached the highest importance to the cost of vaccination. But overall, the COVID-19 vaccine with the higher efficacy, the milder adverse effect, the lower cost, and the longer duration will promote the preference of the public in both countries. Additionally, the public were willing to spend the most money for a reduction in COVID-19 vaccine adverse effect from moderate to very mild (37.476USD for the United States, 140.503USD for China), followed by paying for the 1% improvement in its efficacy and paying for the one-month extension of its duration.

**Conclusion:**

Given the impact of social cues on vaccine acceptance, Chinese government should promote reasonable vaccine-related information to improve national vaccination acceptance. Meanwhile, considering the influence of COVID-19 attributes on public preference and willingness to pay, regulating the vaccine pricing, improving the efficacy of the vaccine, reducing its adverse effect, and prolonging the duration of the vaccine works will contribute to vaccine uptake.

## Introduction

The coronavirus disease 2019 (COVID-19) was reported for the first time in China at the end of 2019 (1), which has caused a global pandemic, threatening the life and safety of all humans and social development (2–4). Studies have shown that the severe acute respiratory syndrome coronavirus 2 (SARS-CoV-2) that caused this pandemic has diversified transmission routes and strong transmission ability that can produce many mild patients in a short period of time (5). Given the susceptible populations and the difficulty of achieving complete isolation of the source of infection, a safe, effective, and broad-spectrum COVID-19 vaccine is widely regarded as one of the most effective means to prevent and control the pandemic.

Due to immune responses generally declining with age (6, 7), the middle-aged and elderly are vulnerable groups in this pandemic (8). According to the preliminary report of 41 cases of the COVID-19 pandemic in Wuhan's Jin Yin-tan Hospital (up to January 2, 2020) (9), the median age of the COVID-19 patients was 49.0 years (range of quartile, 41.0–58.0). Another early case study (10) showed that the COVID-19 incidence was positively correlated with patients' age, especially when aged between 50 and 60. A report by Imperial College London on vaccine distribution strategy (11) found that if the number of vaccines is insufficient and vaccination coverage is low in countries with different income levels and different vaccine distributions, the most effective vaccination strategy should be starting with the high-risk group (vulnerable elderly population) and then moving to the working-age or middle-aged group (12).

Therefore, middle-aged and elderly adults should be regarded as an important target group for vaccination. In January 2021, China launched a “three-step” strategy for vaccination. It is clearly pointed out in the second step that the elderly and other high-risk groups should be vaccinated. Up to March 22, on the premise of fully assessing the health status and risk of infection, some parts of China have begun to carry out COVID-19 vaccination for people over 60 years old who are in good physical condition. However, till Mar 2021, most vaccines still lack a comprehensive review of safety data before marketing and a long-term post-marketing surveillance data in middle-aged and elderly populations (13). Also, with the function of one's immune system attenuating with age, the effectiveness of vaccination will decrease significantly (14). All the above elements may affect the preference and willingness to pay of COVID-19 vaccination in middle-aged and elderly populations.

It is widely known that China and the United States have a large population and numerous confirmed COVID-19 cases (15), so the data samples from the two countries are representative. However, as a developed country, the United States has a certain gap with China as a developing country in terms of many aspects. For example, population and social factors are the most important factors affecting population health. China and the United States are very different in terms of annual income. According to the information released by the US Department of Commerce Census Bureau: In 2020, The “median US-resident household income of $67,500,” The US per capita income and the median per capita income are $38,800 and $27,000, respectively. In 2020, the median per capita disposable income of Chinese residents was 27,000 yuan. In addition, in China, social life is mainly family-oriented and pays more attention to family interests. In the United States, social life is individual-oriented, with more emphasis on independence (16). Moreover, reduced population mobility helped to significantly reduce the COVID-19 transmission capacity, and the socio-economic level (education level) is related to the decrease in the intensity of intra-urban mobility. In the United States, the population mobility changes relatively much in dealing with COVID-19, while the socio-economic status of different Chinese cities is negatively associated with the change in the intensity of intra-urban mobility (17). Therefore, by understanding and comparing the vaccination preferences between the two countries, we can provide reference for each other in epidemic prevention and control. This study aims to investigate attributes of COVID-19 vaccine influencing preference and willingness to pay among Chinese and American middle-aged and elderly adults using a reliable method named discrete choice experiment (DCE).

DCE, as a stated-preference survey method (18), has been widely used to simulate choice behavior by eliciting trade-offs among attributes of hypothetical health services (19), and so on (20), with the advantage of being closer to real-world decision-making. Its theoretical basis, random utility theory (RUT) (21), assumes that respondents generally tend to choose the option with the maximal utility, and its overall utility consisting of two parts, of which the observed characteristics is decomposed according to its attributes in DCE, the unobserved characteristics is random error term (22).

## Materials and methods

### Study design

This study referred to the International Society for Pharmacoeconomics and Outcomes Research (ISPOR) DCE guideline (23) and Lancsar and Louviere's guide (24) to design and implement DCE, so as to evaluate the relative importance of various attributes of the vaccine and the trades-off that respondents are willing to make.

First, a series of vaccine-related attributes and their levels were screened out through a systematic review (25–30). Then, the expert advisory group, including public health scholars and vaccine researching experts, reviewed the content and these attributes with their levels were sorted, classified, and refined. In order to reduce information bias and make the meaning of attributes and levels easier to understand, we added explanations of relevant attributes in the questionnaire design. Based on the above process, six attributes and their levels related to COVID-19 vaccine choices were identified (Table 1).

**Table 1:** Vaccine attributes and their respective levels in this discrete choice experiment.

**Attribute**	**Description**	**Level**
Vaccine varieties	Description: Different kinds of vaccines produced in different countries	Inactivated vaccine^a^
		mRNA vaccine
		Adenovirus vector vaccine
Adverse effect	Severity of side effects after vaccination	moderate^a^
		mild
		very mild
Efficacy	Effectiveness of vaccines to protect vaccines from new coronavirus infection	55%^a^
		65%
		75%
		85%
		95%
Time for the vaccine to start working	The period from the time of vaccination to the time when the vaccine began to work	20 days^a^
		15 days
		10 days
		5 days
The duration of vaccine works	The period from the beginning of the vaccine to its failure	5 months^a^
		10 months
		15 months
		20 months
The cost of vaccination	Cost of the whole process of vaccination	$200^a^
		$150
		$100
		$50
		$0

a Reference level.

Second, the fractional factorial design was used to determine 13 different vaccination scenarios. Owing to the six attributes with three to five levels for each one in our study, organizing levels under different attributes will result in 3,600 combinations (3^2^ × 4^2^ × 5^2^ = 3,600) based on full factorial design, thus increasing cognitive burden on respondents. Therefore, the fractional design was essential in designing the DCE instrument based on the principle of orthogonality, balance and minimal overlap (18). In the DCE design, “orthogonality” means that each attribute should have little to no correlation with other attribute. “Balance” means that each attribute level occurs at the same frequency in an attribute, and “minimal overlap” means avoiding the appearance of the same level for each option in one selection set. In the design process, each selection set includes two types of vaccines and the “Neither” choice. The “Neither” alternative was used to reduce the deviation of the results caused by the subjects being forced to choose (31). An example of the DCE scenario is shown in Table 2.

**Table 2:** An example of choice-based conjoint in the questionnaire.

**Scenario**			
Suppose you are going to get vaccinated, and you are facing several choices of action. Please select the answer that you are most satisfied with?			
**Attributes**	**Vaccine A**	**Vaccine B**	**Neither**
Vaccine verities	Inactivated vaccine	Adenovirus vector vaccines	Neither
Adverse effect	Moderate	Very mild	
Efficacy	85%	75%	
Time for the vaccine to start working	10 days	5 days	
The duration of vaccine works	10 months	15 months	
The cost of vaccination	$0	$50	

Besides DCE, the questionnaire home page introduced general information about COVID-19 and its vaccination. Furthermore, we also collected demographic information of the respondents, including gender, highest educational level, country, occupation fields and annual income level. Additionally, respondents were asked to rate their acceptance of vaccination, with and without recommendations from friends, family members or employers (the social cues referred to in our study). The detailed questions were “How do you evaluate your willingness and acceptance to be vaccinated?” and “How do you rate your acceptance if your friends, family, employers, etc. suggest that you do so?”. The answer was set to a scale of “totally unwilling 0” to “totally willing 10.” Respondents with an answer score of more than six were defined as positive acceptance (32).

### Data collection

An anonymous self-administered cross-sectional survey was done online globally from January 28, 2021, to February 27, 2021. The questionnaire was conducted using Lighthouse Studio (version 9.12.0, Sawtooth Software, Orlando, FL, USA). The College Review Committee approved the study of Jinan University. Middle-aged and elderly respondents (41 and above) from China and the US who had no cognitive impairment were included in our study. In China, according to the principle of multi-stage stratified cluster sampling, based on geographical division and population distribution, 2 provinces were selected from each of 7 administrative regions (East China, South China, North China, Central China, Southwest, Northeast and Northwest China) by random number table method. Then the random number table method was utilized to select 3–4 cities from the selected provinces, and skipped this step if they are municipalities directly under the central government. Questionnaires were distributed through investigators. Two investigators were recruited in each city. Before the formal distribution of questionnaires, investigators were trained uniformly, and each investigator was responsible for collecting 50–70 questionnaires. In the US, MTurk was used to recruit questionnaire fillers, ensuring that the data were representative of most psychological dimensions of the general population (33).

During the distribution of questionnaires, respondents were required to offer a randomly generated code after completing the questionnaire in order to ensure that they were real people. If the website of the questionnaire was closed before completion, no data would be stored. The study was approved by the Jinan University Medical Ethics Committee (JNUKY-2021-004).

According to the rule of thumb as proposed by Johnson (34), the minimum sample size of DCE study could be calculated with the formula of:


$$\begin{array}{l} N\;>\;\frac{500c}{\left(t\times a\right)} \end{array}$$

where *N* is the recommended minimum sample size, *t* is the number of selection sets, *a* is the number of selections per set, and *c* is the maximum number of attribute levels. In our study, *t* is 13, *a* is 3, and *c* is 5, so *N* needed to be more than 64.10. Considering that there may be some invalid questionnaires, we set the sample size at 100 respondents each country.

### Data analysis

IBM SPSS Statistics 26.0 (IBM Corporation, Armonk, NY, USA) and Lighthouse Studio (version 9.12.0, Sawtooth Software, Orlando, FL, USA) were applied for statistical analysis. The characteristics of demographic information was summarized descriptively. Given that confounding factors of baseline characteristics may cause potential biases (35, 36), propensity score matching (PSM) was required to adjust for variables affecting respondents' choices (37), thus helping strengthen causal inferences in observational studies (38). In our study, the outcome variables which propensity scores calculated for were “self-reported acceptance of COVID-19 vaccines with social cues' and ‘self-reported acceptance of COVID-19 vaccines without social cues.” The determinant in our study was the country of the respondents, i.e., China or the US. Through literature review, we found that men and women will affect their acceptance, adverse reactions and immune effects of vaccines due to differences in hormones, genetics and environment (39, 40). People with higher education level have higher awareness of vaccine knowledge, stronger awareness of disease prevention, and are more inclined to prevent disease through vaccination (41, 42). Groups with low income usually have low awareness of vaccines, generally lack trust in newly launched COVID-19 vaccines, and are unwilling to vaccinate or pay for them (43, 44). Therefore, the selected variables considered as possible confounders were gender, highest educational level, and annual income. They were encoded as dummy variables (e.g., respondents whose annual income under 20,000 USD per year were coded as “0” and those with 20,000 USD per year and above were coded as “1”).

To analyze the results of the DCE, a conditional logit model (CLOGIT) with dummy-coded levels for each attribute was used, and one specific level in each attribute was selected as the reference level. The calculation outcome includes coefficient, *P*-value, odds ratio (ORs), 95% confidence intervals (95%CI), where the magnitude of the coefficients indicates the size of the impacts of attributes on utility, and the sign of coefficients reflects whether the effect is positive or negative.

For the willingness to pay (WTP), the coefficient can be used to estimate the WTP for non-monetary attributes. WTP indicates how much a person is willing to pay for the unit change of preference attributes. It can be estimated by calculating the ratio of other attribute's coefficient over the cost attribute coefficient (see Data Sheet 1 for equations). For the interpretation of the results, negative currency values refer to the amount that respondents were willing to pay for another level.

## Results

### General PSM results

**Figure 1:**
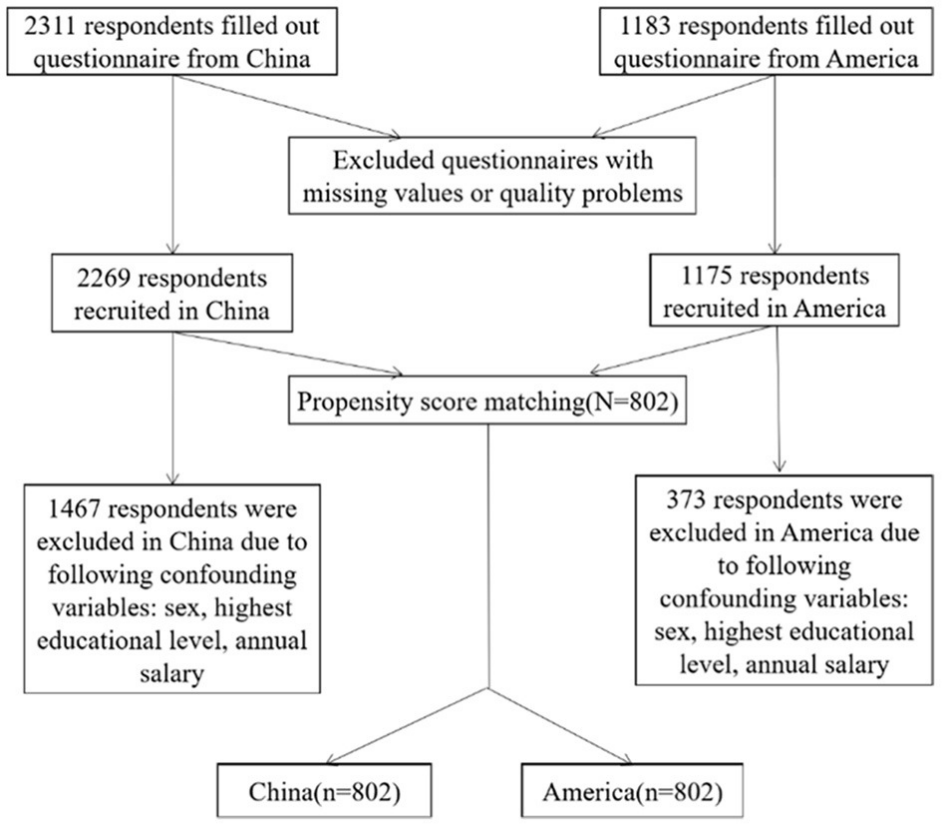
The procedure of propensity score matching.

A total of 3,494 respondents with 2,311 from China and 1,183 from the US completed the questionnaire, of which 50 were excluded (5 due to choosing “other” options on gender question and 45 for missing the annual income information). Three thousand four hundred forty-four responses were valid and included in our study, yielding a 98.6% effective rate. Of the 3,444 respondents, 1,604 respondents with 802 from the US and 802 from China went through the PSM. The PSM procedure is shown in Figure 1.

The differences of demographic characteristics between the respondents from the US and China before and after PSM are presented in Table 3. We could distinguish from the *P*-value that the matching method achieved balance in selected covariates between the two groups. For example, the differences in respondents' gender between the two countries reached conventional levels of statistical significance (*P* < 0.001) before matching, while it was not significant after matching (*P* = 0.364) (Table 3).

**Table 3:** Demographic characteristics of non-matched and propensity score-matched respondents.

**Baseline matching characteristics**	**Non-matched respondents**			**Propensity score-matched respondents**		
	**China (*****n*** = **2,269)** ***n*** **(%)**	**USA (*****n*** = **1,175)** ***n*** **(%)**	***P-*** **value**	**China (*****n*** = **802)** ***n*** **(%)**	**USA (*****n*** = **802)** ***n*** **(%)**	***P-*** **value**
**Gender**			< 0.001			0.364
Male	1,107 (48.8)	494 (42.0)		353 (44.0)	335 (41.8)	
Female	1,162 (51.2)	681 (58.0)		449 (56.0)	467 (58.2)	
**Highest education level**			< 0.001			0.999
Pre-primary education or primary school education to Vocational school education	1,587 (69.9)	372 (31.7)		372 (46.4)	372 (46.4)	
Bachelor's degree (or other same-level degrees) to PhD degree	682 (30.1)	803 (68.3)		430 (53.6)	430 (53.6)	
**Annual income**			< 0.001			0.999
Under 20,000 USD per year	1,635 (72.1)	241 (20.5)		241 (30.0)	241 (30.0)	
20,000 USD per year and above	634 (27.9)	934 (79.5)		561 (70.0)	561 (70.0)	

### “Self-reported” acceptance of COVID-19 vaccine

**Figure 2:**
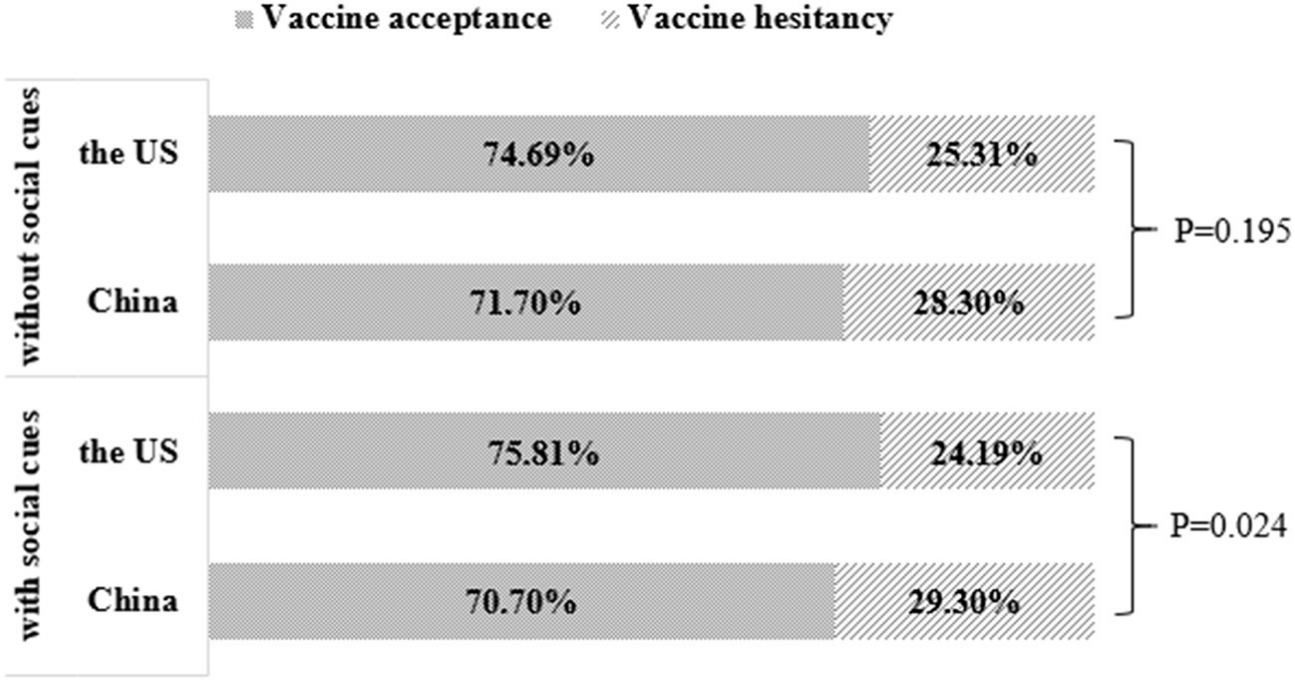
COVID-19 vaccination acceptance comparison between China and the US depending on social cues.

The results showed that the acceptance rate of the COVID-19 vaccine among the Chinese aged 41 years and above was 71.7% (without social cues) and 70.7% (with social cues). The corresponding data of the US was 74.7% (without social cues) and 75.8% (with social cues). Chi-square test showed that without social cues, there was no significant difference in self-reported acceptance of COVID-19 vaccine between the two countries' respondents. In the case of social cues, there was a significant difference in self-reported attitudes toward vaccination between the two countries' respondents. Further comparison found that the social cues in the US increased the self-reported acceptance to be vaccinated, while the social cues in China did the opposite (Figure 2).

### Relative importance of the COVID-19 vaccine attributes and their levels

**Figure 3:**
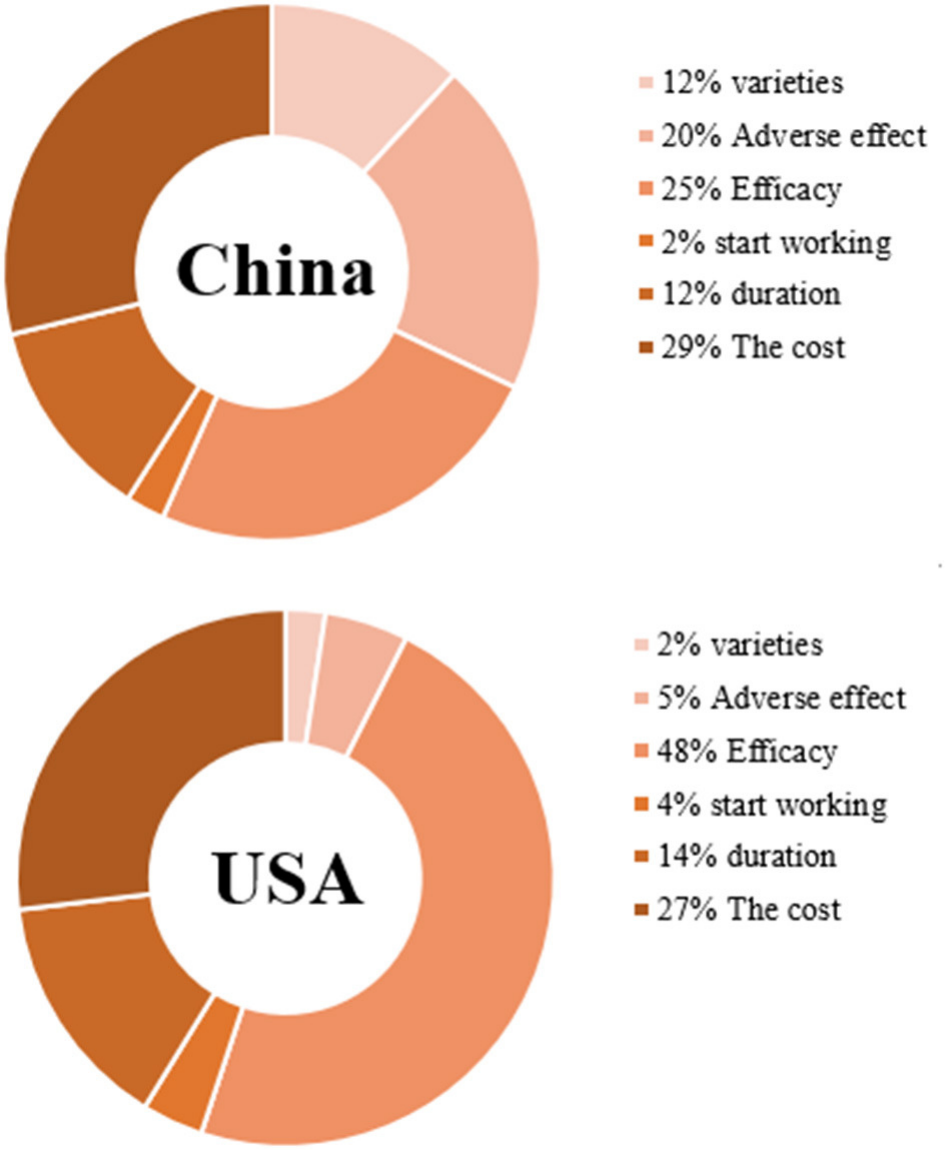
Attribute relative importance in a conditional logit model.

Tables 4, 5 show the CLOGIT estimation results of Chinese and the US respondents' relative importance for each attribute and its level of the COVID-19 vaccine. Among them, the efficacy of the COVID-19 vaccine had the greatest impact on the choice of the American public. They were more willing to choose the COVID-19 vaccine with higher efficacy. A vaccine with 95% efficacy was 6.40 times (95%CI: 5.97–6.85) more likely to be selected than one with 55% efficacy. Comparatively speaking, the cost of vaccine had the greatest influence on the choice of the Chinese public. They preferred to choose the free COVID-19 vaccine (coefficient = 0.44, *P* < 0.001), and the probability of choosing a vaccine with $0 was 2.02 times (95%CI: 2.65–3.06) higher than one with $200. And the same for the respondents from two countries was that the vaccine with very mild adverse effect is more acceptable than the vaccine with moderate adverse effect (the US: OR 1.22, 95%CI 1.16–1.28; China: OR 1.64, 95%CI 1.57–1.71). Also, the vaccine with the duration of 20 months was more acceptable than the vaccine with the duration of 5 months (the US: OR 1.75, 95%CI 1.65–1.85; China: OR 1.35, 95%CI 1.28–1.42). As for the order of importance of each attribute, the US: the efficacy of the vaccine (47.64%) > the cost of vaccination (26.87%) > the duration of vaccine works (14.36%) > adverse effect of the vaccine (5.04%); China: the cost of vaccination (28.76%) > the efficacy of the vaccine (24.57%) > adverse effect of the vaccine (20.20%) > the duration of vaccine works (12.24%). The influence weight of all attributes is shown in Figure 3.

**Table 4:** The US conditional logit model analysis results (*n* = 802).

**Label**	**Coefficient**	**Std error**	**t Ratio**	***P*-value**	**Odds ratio**	**95%CI**	
**Vaccine varieties**							
Inactivated vaccine	−0.05	0.02	−2.07	0.039	Reference		
Adenovirus vector vaccines	0.01	0.02	0.39	0.699	1.06	1.01	1.12
mRNA	0.04	0.02	1.73	0.084	1.10	1.05	1.15
**Adverse effect**							
moderate	−0.12	0.02	−4.86	< 0.001	Reference		
mild	0.04	0.02	1.76	0.078	1.18	1.12	1.24
very mild	0.08	0.03	3.04	0.002	1.22	1.16	1.28
**Efficacy**							
55%	−0.93	0.05	−20.18	< 0.001	Reference		
65%	−0.62	0.04	−16.53	< 0.001	1.37	1.27	1.47
75%	0.10	0.04	2.68	0.008	2.81	2.62	3.03
85%	0.54	0.03	16.68	< 0.001	4.36	4.09	4.64
95%	0.92	0.03	26.52	< 0.001	6.40	5.97	6.85
**Time for the vaccine to start working**							
20 days	−0.04	0.03	−1.14	0.256	Reference		
15 days	0.04	0.03	1.39	0.166	1.08	1.02	1.14
10 days	−0.07	0.03	−2.38	0.017	0.96	0.91	1.02
5 days	0.07	0.03	2.26	0.024	1.11	1.05	1.18
**The duration of vaccine works**							
5 months	−0.31	0.03	−9.10	< 0.001	Reference		
10 months	−0.09	0.03	−2.88	0.004	1.25	1.17	1.32
15 months	0.15	0.03	4.86	< 0.001	1.58	1.49	1.68
20 months	0.25	0.03	8.47	< 0.001	1.75	1.65	1.85
**The cost of vaccination**							
$200	−0.44	0.04	−10.83	< 0.001	Reference		
$150	−0.26	0.04	−7.17	< 0.001	1.19	1.11	1.28
$100	−0.10	0.03	−2.98	0.003	1.41	1.32	1.50
$50	0.20	0.03	5.87	< 0.001	1.89	1.77	2.02
$0	0.61	0.04	16.41	< 0.001	2.85	2.65	3.06

**Table 5:** China conditional logit model analysis results (*n* = 802).

**Label**	**Coefficient**	**Std error**	**t Ratio**	***P*-value**	**Odds ratio**	**95%CI**	
**Vaccine varieties**							
Inactivated vaccine	0.16	0.02	7.39	< 0.001	Reference		
Adenovirus vector vaccines	−0.13	0.02	−5.90	< 0.001	0.75	0.72	0.78
mRNA	−0.03	0.02	−1.44	0.15	0.83	0.79	0.86
**Adverse effect**							
Moderate	−0.28	0.02	−12.22	< 0.001	Reference		
Mild	0.06	0.02	2.64	0.01	1.40	1.34	1.46
Very mild	0.22	0.02	9.81	< 0.001	1.64	1.57	1.71
**Efficacy**							
55%	−0.32	0.04	−8.87	< 0.001	Reference		
65%	−0.22	0.03	−7.20	< 0.001	1.10	1.04	1.17
75%	0.09	0.03	2.64	0.01	1.51	1.41	1.62
85%	0.18	0.03	5.83	< 0.001	1.65	1.55	1.75
95%	0.28	0.03	8.62	< 0.001	1.82	1.71	1.94
**Time for the vaccine to start working**							
20 days	0.01	0.03	0.24	0.81	Reference		
15 days	−0.03	0.03	−1.07	0.29	0.97	0.92	1.02
10 days	0.03	0.03	1.08	0.28	1.02	0.97	1.08
5 days	−0.01	0.03	−0.26	0.79	0.99	0.93	1.04
**The duration of vaccine works**							
5 months	−0.17	0.03	−5.54	< 0.001	Reference		
10 months	−0.04	0.03	−1.54	0.12	1.13	1.08	1.20
15 months	0.08	0.03	2.72	0.01	1.28	1.21	1.35
20 months	0.13	0.03	4.85	< 0.001	1.35	1.28	1.42
**The cost of vaccination**							
$200	−0.26	0.04	−7.27	< 0.001	Reference		
$150	−0.22	0.03	−6.71	< 0.001	1.04	0.97	1.11
$100	−0.04	0.03	−1.40	0.16	1.25	1.18	1.32
$50	0.09	0.03	2.85	0.00	1.42	1.34	1.50
$0	0.44	0.03	13.00	< 0.001	2.02	1.89	2.16

### Willingness to pay

The WTP results of the public in both countries is shown in Table 6. Respondents were willing to spend the most money for a reduction in COVID-19 vaccine adverse effect from moderate to very mild, and the WTP of the Chinese (140.503USD) was higher than that of the Americans (37.476USD). Also, respondents were willing to pay more for the vaccine with higher efficacy (in the US, if the efficacy of the vaccine increased by 1%, their willingness to pay would increase 8.863USD; in China, if the efficacy of the vaccine increased by 1%, their willingness to pay would increase 4.272USD). Additionally, the extension of the duration of vaccine works is worth costing more money by the respondents from the two countries (in China, their willingness to pay would increase by 5.676 USD for each month of duration extension; in the US, their willingness to pay would increase by 7.124 USD for each month of duration extension). However, in terms of vaccine variable attribute, the Chinese public were more willing to pay for inactivated vaccine, while the American public were more willing to spend more money in mRNA vaccine and adenovirus vector vaccine. Of note, one finding was that the Chinese public were willing to pay more for the vaccine that have a longer time to start working.

**Table 6:** Respondents WTP^a^ in the US and China.

**Attribute**	**Overall WTP in the US (*N* = 802) (USD)**	**Overall WTP in China (*N* = 802) (USD)**
**Vaccine variable**		
Adenovirus vector vaccines	−11.653	82.597
mRNA vaccine	−17.858	54.436
**Adverse effect**	−37.476	−140.503
**Efficacy**	−8.863 (per % increase)	−4.272 (per % increase)
**Time for the vaccine to start working**	1.375 (per day increase)	−0.272 (per day increase)
**The duration of the vaccine**	−7.124 (per month increase)	−5.676 (per month increase)
**The cost of vaccination**	Reference	Reference

a WTP, willingness to pay.

## Discussion

This study compared the similarities and differences of public acceptance, preference and willingness to pay for the COVID-19 vaccine between China and the US. And the reason for choosing these 2 countries for comparison is that they are major countries of vaccine research and development (45). When COVID-19 vaccination was carried out in early 2021, the investigation of people's preference for vaccine attributes and levels would help to increase the vaccination rate. Additionally, in the face of the outbreak, the United States aimed to herd immune focus on severe cases, and China's “five early” policy, namely “early detection, early report, early investigation, early isolation, early treatment,” strive to completely block the spread of COVID-19. The two countries and the current vaccination rate is very different. Therefore, understanding vaccination preference for acceptance and vaccination rate is crucial. Moreover, during our investigation, the pandemic situation in China showed an improving trend, while the pandemic situation in the United States is worsening. So, we can further compare the public preferences and willingness to pay for vaccines under the two countries' different pandemic situations.

In terms of acceptance, the willingness of both Chinese and American respondents to receive COVID-19 vaccine has reached 70%. The author believed that this was related to the susceptibility of COVID-19 (46). COVID-19 has a strong transmission ability, and it is difficult to completely isolate the source of infection. Therefore, the public generally hopes to generate immunity through vaccination. However, the “self-reported” vaccine acceptance of the American public was higher than that of the Chinese public. The author held that this difference may be caused by the perceived health risks of the public (47, 48). Neumann-Bohme et al. (49) found that people in areas seriously affected by the pandemic have a higher willingness to be vaccinated against COVID-19. Although China was the first country to encounter the COVID-19 pandemic, it has quickly and effectively controlled the pandemic by taking many measures, such as active case surveillance and management, community screening, isolation of medical personnel, and centralized deployment of resources. The public has returned to normal life and work, resulting in a reduction in the perceived health risks of the public. But for the US, the number of people infected with COVID-19 was still large. Due to panic and concern about the severity of the disease, the perceived health risks of the American public were also be higher. Therefore, in the United States, a higher proportion of the public were willing to receive the COVID-19 vaccine. Additionally, under the influence of social clues, the Chinese public's acceptance of vaccines declined slightly, while the United States was on the contrary. The author explained that the information environment built by the media and the health system was an important factor affecting vaccine hesitancy (50). Research showed that in China, those who relied on health care providers, social media, the Internet and family/friends as the main information source about COVID-19 tended to express higher negative vaccine intent (51, 52). As for the United States, many articles on factors related to vaccine hesitancy were published in 2020 (53, 54), which served as a warning and to some extent helped the social media to carry out more targeted vaccination publicity. Therefore, the survey data from the United States is consistent with Larson et al. (55), indicating that other people's encouragement and suggestions were helpful for vaccination.

The CLOGIT results showed that the American public attached most importance to the efficacy of the vaccine, followed by the cost of vaccination. Comparatively speaking, the cost of vaccination had the greatest impact on the Chinese public's choice of COVID-19 vaccine, followed by the efficacy of the vaccine. It can be seen that the efficacy of the vaccine and the cost of vaccination were the two factors that the respondents of both countries attach the most importance to when choosing. The result was in line with the finding of Williams et al. (56) that the most common reasons for public hesitation to accept the vaccine include doubts about the efficacy of the vaccine. At the same time, the attribute preference was to some extent consistent with the conclusion of the early survey report in the US, which found that the cost of vaccination was a concern to Americans (54). Another review also showed that 49% respondents expected the vaccine to be free (paid for by insurance or the government) (57). In addition, the third important attribute of the US respondents concern was the duration of vaccine works, while the third important attribute of Chinese respondents concern was the adverse effects of vaccines. As for the differences in the ranking of attribute importance between the public of the two countries, the author believed that it can be explained according to the different social backgrounds of the two countries at that time (51). During the period of study, the United States was in the pandemic situation, and the public perceived high health risks, so they paid more attention to the efficacy and duration of the vaccine, hoping that vaccination could help them build immune defense. Whereas, the pandemic situation in China tended to be stable, and the perceived risk of disease was low. Therefore, people paid more attention to the price and safety of vaccines, and did not want to pay for vaccination or be affected by adverse effects. In terms of vaccine varieties, the American public was more inclined to vaccinate with mRNA vaccine, while the Chinese public was more inclined to vaccinate with inactivated vaccine. Previous studies have shown that the incidence of adverse reactions of different varieties of COVID-19 vaccines is roughly as follows: adenovirus vector vaccine>mRNA vaccine>inactivated vaccine (58). From the above effect, we could infer that Chinese public's choice of inactivated vaccines was consistent with their psychology that they do not want to be affected by the adverse effects of vaccines. Moreover, the preference for vaccine varieties was also related to its availability (47), that is, the reality of vaccine supply in different countries. In the United States, COVID-19 vaccine mainly includes the mRNA produced by Pfizer and the adenovirus vector vaccine produced by Jassen, while in China, the inactivated vaccines of Sinopharm and Sinovac are the main ones. Therefore, the American public has received more information about mRNA and adenovirus vector vaccines, while the Chinese public has received more information about inactivated vaccines, which also led to different preferences for vaccine varieties between the two countries' respondents. The further analysis of each attribute level shows that COVID-19 vaccine with higher efficacy, lighter adverse reactions, lower cost and longer duration is generally preferred by the public in both countries. Concerning WTP, the respondents in both countries were willing to spend more money in reducing the adverse effect of the vaccine rather than get a more efficient vaccine, suggesting that although the public attached most importance to an efficient vaccine, they were still more worried about the adverse effect of the vaccine than the efficacy of the vaccine. Concretely, to reduce adverse effect from moderate to very mild, the WTP of the Chinese (140.503USD) was higher than that of the Americans (37.476USD). One previous study (59) in Chongqing showed that more than 25% respondents were willing to pay $50 for the COVID-19 vaccine and over 5% respondents were willing to pay $250 for the COVID-19 vaccine. And another study (60) reported that the average out-of-pocket WTP for full COVID-19 vaccination was $36.8. To the US, Reiter et al. (61) found that only 35 percent of the participants would be willing to pay $50 or more for the COVID-19 vaccine. Considering the heterogeneity of demographic characteristics and the inconsistence of questionnaire distribution time, there were some differences in respondents' willingness to pay in different studies. Since the pandemic condition and information about the vaccine might change over time, further study may be required to monitor the trend worldwide.

With regard to the differences in public's willingness to pay for vaccine varieties between the two countries, the author thought that it was related to social resources. In the US, the COVID-19 vaccine mainly included mRNA produced by Pfizer and adenovirus vector vaccine produced by Jassen, while in China, the inactivated vaccines of Sinopharm and Sinovac are the main ones. As for why the Chinese public were willing to pay more for the vaccine that have a longer time to start working, the author speculated that the Chinese public might think that the vaccine with a short time to start working was hastily put on the market without sufficient clinical trials, so they had doubts about its safety and adverse effects, thus unwilling to support it.

### Strengths and limitations

After the screening of sample, the respondents of the two countries had no significant difference in gender, highest education level, and annual income, which can better rule out the influence of demographic characteristics on vaccine attribute selection. In addition, each attribute was explained in detail in the DCE design process, which ensured that the meaning of the question could be understood. In a selection set, the “Neither” option was provided, which reduced the risk of overestimating the influence of an attribute. And the rational use of the conditional logit model could reasonably show the public preference for COVID-19 vaccine in China and the US.

Meanwhile, the study has some limitations. First, in view of the dynamic and variable vaccination rate and acceptance, the results of the cross-sectional study could only reflect the current situation and could not make the analysis of long-term causality. Second, this study investigated the participants' self-reported acceptance to be vaccinated, which may be different from the actual situation of “vaccine acceptance.” Third, because this survey was carried out at the initial stage of the launch of the vaccine, the main purpose was to understand the preference weights of the middle-aged and elderly groups for each attribute of COVID-19 vaccine as soon as possible, so other relevant factors were not taken into account, which led to limited information reflected in the data. However, the data in this paper still have some value. Middle-aged and elderly people were vulnerable groups in this pandemic. By clarifying their acceptance and preference at the beginning of vaccine promotion, the government will carry out more targeted work related to vaccination. Finally, the data in this article reflected the public's choices when the COVID-19 vaccine was at its initial stage of listing and promotion. So, the results cannot be extrapolated to the public's analysis of effective vaccine preference for Delta and Omicron strains. Considering that the research and development of COVID-19 vaccine will continue to progress over time, the interpretation of the results should still be cautious.

## Conclusion

In summary, there were three main findings in our study. First, the public acceptance of the vaccine in China and the US accounted for more than half, and the social cues in the US increased the self-reported acceptance, while the social cues in China did the opposite. Second, the efficacy of COVID-19 vaccine and the cost of vaccination were the most important attributes among respondents from two countries, and vaccine with the higher efficacy, the milder adverse effect, the less cost, and the longer duration will promote the preference of the public in both countries. Third, the public were willing to spend the most money for a reduction in COVID-19 vaccine adverse effect from moderate to very mild. Based on the above results, we suggested that the two countries' governments, especially the one in China, should correct peoples' improper view on COVID-19 vaccine and strengthen the publicity of vaccination to further reduce vaccine hesitancy. Meanwhile, scientists and pharmaceutical companies of the two countries should work together to improve the efficacy of the vaccine, reduce its adverse effect, and prolong its duration. Moreover, it also of great importance to regulate the pricing of the vaccine. Only combined with the above joint efforts, can we enable the middle-aged and elderly adults from China and the US to better vaccinate, thus establishing mass immunity.

## Data availability statement

The original contributions presented in the study are included in the article/Supplementary material, further inquiries can be directed to the corresponding author.

## Ethics statement

The studies involving human participants were reviewed and approved by the study was conducted according to the guidelines of the Declaration of Helsinki, and approved by the Jinan University Medical Ethics Committee (JNUKY-2021-004). The patients/participants provided their written informed consent to participate in this study.

## Author contributions

ZH and TL were actively involved in DCE design. WY was responsible for data acquisition and screening. XL and TL analyzed data. XL interpreted the results of the data analysis. XL, LYa, GT, and BF drafted the manuscript. XL, XJ, XZ, and MH revised the manuscript. LYu and W-KM revised the manuscript critically for important intellectual content, and then approved the version to be published. All authors have read and approved the manuscript.
