# Supplementary material for: Understanding influencing attributes of COVID-19 vaccine preference and willingness-to-pay among Chinese and American middle-aged and elderly adults: A discrete choice experiment and propensity score matching study
Source: Front Public Health. 2023 Mar 16;11:1067218. doi: 10.3389/fpubh.2023.1067218 (PMC10060971; doi:10.3389/fpubh.2023.1067218)
Supplement: Supplementary file 1 [file Data_Sheet_1.pdf]

Among them, β_{cost} is the marginal utility of income, and the method of its calculation in our study is shown below. To continuous variables, the calculation of WTP is shown in the Equation 1. To categorical variables, the calculation of WTP is shown in the Equation 2. When analyzing the results, negative currency values refer to the amount that respondents were willing to pay for another level, and the greater the negative value, the more willing the respondents are to pay for the change of the attribute level.

$$\beta_{cost} = \frac{\text{Effect coefficient}_{\$0} - \text{Effect coefficient}_{\$200}}{\$0 - \$200}$$

$$\text{WTP} = \frac{(\text{Effect coefficient}_{\text{specified level}} - \text{Effect coefficient}_{\text{reference level}}) / \triangle \text{Level}_{\text{attribute}}}{\beta_{cost}} \quad (1)$$

$$\text{WTP} = \frac{\text{Effect coefficient}_{\text{specified level}} - \text{Effect coefficient}_{\text{reference level}}}{\beta_{cost}} \quad (2)$$
